# An integrated synchronization approach for studying cell-cycle dependent processes of mammalian cells under physiological conditions

**DOI:** 10.1186/1753-6561-7-S6-P16

**Published:** 2013-12-04

**Authors:** Oscar B Platas, Uwe Jandt, Volker Sandig, Ralf Pörtner, An-Ping Zeng

**Affiliations:** 1 Institute of Bioprocess and Biosystems Engineering, Hamburg University of Technology, Hamburg, D-21073, Germany; 2 ProBioGen AG, Berlin, D-13086, Germany

## Introduction

The study of central metabolism and the interactions of its dynamics with growth, product formation and cell division is a key issue for decoding the complex metabolic network of eukaryotic cells. For this purpose, not only the quantitative determination of key cellular molecules is necessary, but also the variation of their expression rates in time, e.g. during cell cycle. The enrichment of cells within a specific cell cycle phase, referred to as cell synchronization, and their further cultivation allow for the generation of a cell population with characteristics required for cell cycle related dynamic studies. Unfortunately, most of the synchronization methods used are not suitable for study under unperturbed physiological conditions.

Physical selective methods appear to be a better choice. Among them, the method of countercurrent centrifugal elutriation allows for an efficient separation of different cell subpopulations from an asynchronous cell population according to the cell size. Within an elutriated cell subpopulation high similarity in the size and DNA content of the cells can be achieved. Given the reproducibility of this method, high cell numbers can be obtained for inoculation of controlled bench-top bioreactors with synchronous cells. By integration of this method for synchronous cell generation and a culture method for further unperturbed growth, sampling of synchronous cells can be performed over many synchronous population doublings.

## Materials and methods

Using the combined approach mentioned above, centrifugal elutriation was employed for synchronization in different cell cycle phases of the industrial human cell line AGE1.HN^® ^(ProBioGen AG, Berlin, Germany) and a CHO-K1 cell line (CeBiTec, Bielefeld, Germany). Cells were cultivated in bench-top bioreactors with culture volumes ranging between 200 mL and 1 L. A dialysis bioreactor (Bioengineering AG, Switzerland) with a total volume of 3.8 L was used for the cultivation of one cell line in order to allow for a higher number of synchronous cell divisions. In this bioreactor cells are separated from the conditioning chamber, where pH and DO control takes place. In this way cells can't be damaged neither by increase in stirring rate nor by bubble sparging. Furthermore, continuous nutrient exchange takes place through the dialysis membrane. Cell density values of 4.2 × 10^7 ^cells mL^-1 ^have been reached in this system with AGE1.HN^® ^cells without noticeable change in the cell specific growth rate.

## Results

Our first results had already demonstrated the successful separation of a heterogeneous AGE1.HN^® ^cell population into synchronous subpopulations [1]. Independently of the targeted cell cycle phase, the countercurrent centrifugal elutriation allowed for a reproducible and scalable cell synchronization of AGE1.HN and CHO-K1 cells with high synchrony degrees, up to 95% in G_1_, 53% in S and 75% in the G_2_/M phases.

**Figure 1:**
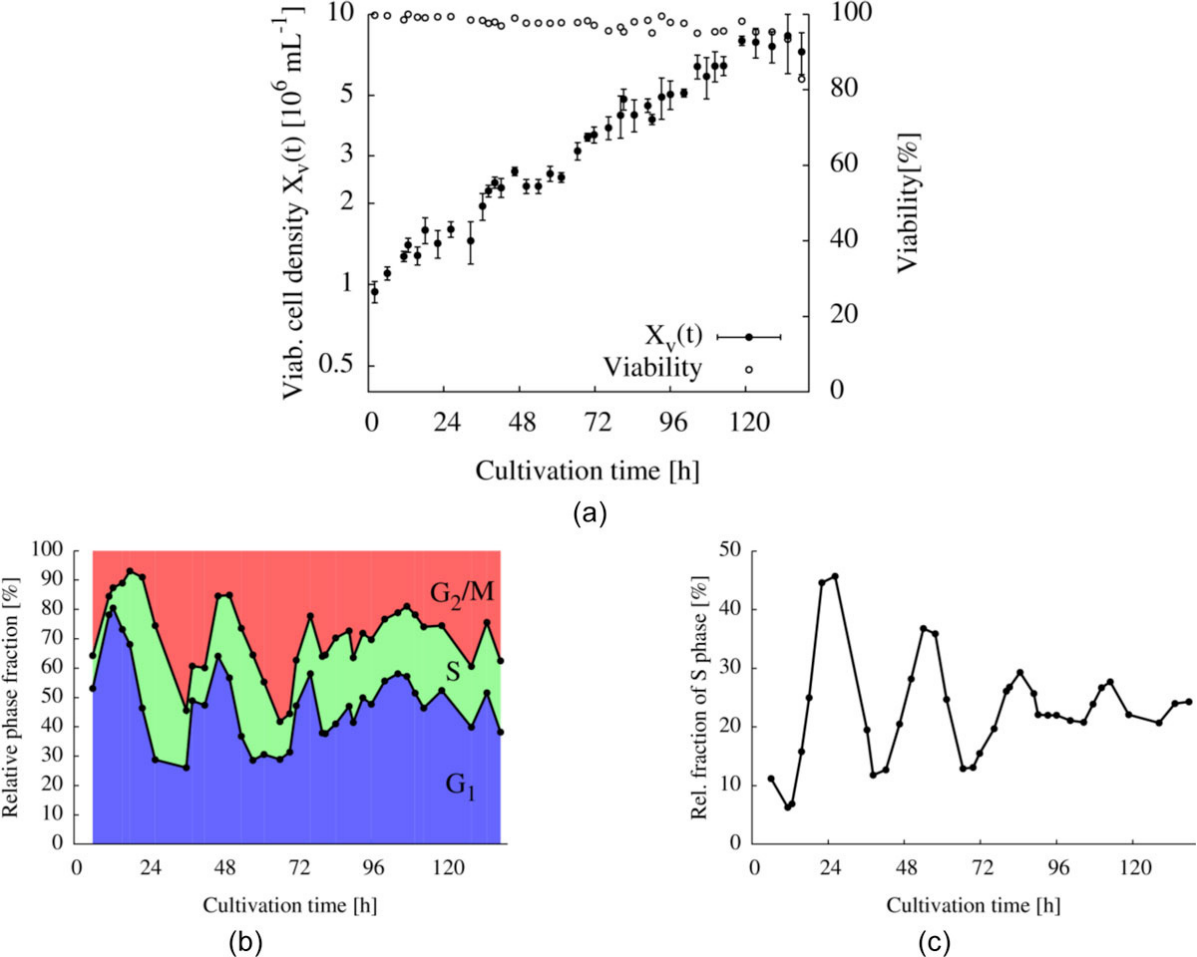
**Synchronous growth of AGE1.HN cells in a dialysis bioreactor**. The cultured cells were elutriated with high synchrony in the G_2_/M phase. **(a): **viable cell density and viability, **(b): **percentage values of the cell cycle phase distribution, **(c): **distribution of the S phase, exhibiting a damped oscillation.

After assessing the reproducibility of elutriation results, the process was scaled up successfully for inoculation of a dialysis bioreactor, where synchronous unperturbed growth was observed at least for 4 cell divisions (Figure 1). A very clear damped oscillation of the cell cycle phases could be observed during synchronous growth (Figure 1b and 1c). Moreover, a sawtooth-like oscillation of the cell diameters confirmed the successful synchronous growth of the cells. Bioreactor culture showed no noticeable perturbation in the doubling time of the population.

## Conclusions

With these results, one of the most important requirements for population-based research of mammalian cells was fulfilled. The dynamic behaviour of the synchronous growing cells was systematically studied not only based on cell growth, but also on the distribution of the cell size and the DNA content of the cells. Furthermore, dialysis culture allowed for a higher number of synchronous cell divisions without noticeable perturbations. With this contribution, we present an integrated approach for cell synchronization and further unperturbed cultivation which is useful for studying cell-cycle dependent processes under physiological conditions.
